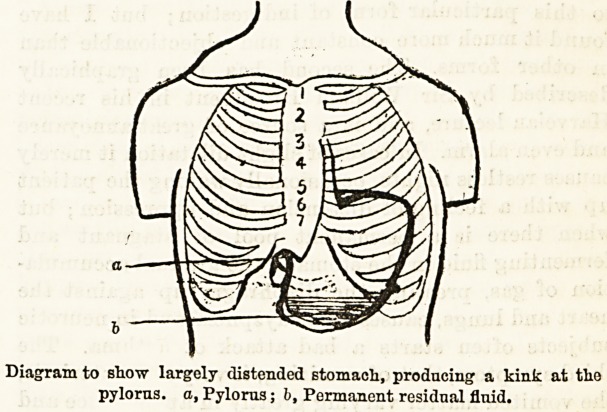# Dilatation of the Stomach

**Published:** 1894-04-14

**Authors:** Walter L. Woollcombe

**Affiliations:** Assistant Surgeon South Devon Hospital, Plymouth


### DILATATION OF THE STOMACH.

Walter L. Woollcombe, F.R.C.S.E., Assistant Surgeon South Devon Hospital, Plymouth.

In a short paper like the present it is impossible to discuss more than one variety of the huge subject of dyspepsia. I shall, therefore, confine my remarks to that particular form which results in dilatation of the stomach, a form of which I have seen and treated a considerable number of cases, and which would seem to be a more common sequel of indigestion in Devon and Cornwall than the writings of other investigators would lead us to believe is the case in other parts of England. Whether this is the result of the copious draughts of cider which farm labourers habitually take during harvesting or whether it is the result of the emotional and neurotic tendency of the inhabitants I do not pretend to say, but I incline to the latter view. We may most conveniently consider the subject under the following heads: (1) Causes; (2) Symptoms and Diagnosis; (3) Treatment.

1. *Causes.*—These may be roughly divided into (*a*) mechanical, and (*b*) nervous, as long as it is under-

Diagram showing healthy stomach after moderate meal.

stood that no absolutely hard and fast rule can be laid down. A case which perhaps was started by mechanical causes may, by the time it comes under observation, have taken on so many of the characteristics of the second class as to make it impossible to assign it to one or the other with certainty.

(*a*) *Mechanical.*—In considering this class, I will at once put aside those cases which are obviously the result of some organic stricture of the pylorus, either due to carcinoma or gumma, and confine my remarks simply to those due to sudden and over distension. There can be no doubt that this condition frequently

Diagram to show largely distended stomach, producing a kink at the pylorus. *a*, Pylorus; *b*, Permanent residual fluid.

obtains, and may be produced by either solids or liquids, but more commonly by some starchy foods, such as bread or potatoes, which, after ingestion, continue to swell. The stomach is enormously blown out, and prevented from expelling its contents by either temporary paralysis of its muscular walls from overstretching, or from temporary pyloric obstruction owing to a kink being produced at that point by the heavy stomach dragging downwards, and the pylorus being fixed by a short lesser omentum. One can readily imagine that several repetitions of this state of things would so weaken the muscular walls and blunt the sensibility of the mucous membrane that a state of permanent dilatation must result and the stomach be converted into a pond of stagnant fluid.

(*b*) The nervous causes act in an entirely different way, and produce their effects by starving the stomach muscle of its proper supply of nerve impulse. Perhaps the most common causes are (1) those which produce an enfeeblement of the whole nervous system, such as anæmia, alcoholism, &c.; (2) those which divert that portion of nerve supply which should be devoted to the stomach into some other channel, such as overwork, sitting down to meals thinking of other things, and rushing off directly they are swallowed and devoting all mental power to some other subject. Under either of these conditions it is obvious that the stomach has not a fair chance, and unless the food which is taken is of a very easily assimilable nature it is retained as an irritant. This is usually the case, as the particular food taken is the result of some passing fancy, and not chosen because of its easy digestibility, added to which it is bolted in a hurry without sufficient mastication. The result of this enervation, from whichever cause, is a gradual yielding and thinning of the stomach walls. So much for the more common modes of causation. We will now glance briefly at

2. *Symptoms.*—And, in doing so, I shall pass over the numberless subjective symptoms which are complained of in most cases of indigeston, and which, therefore, often form part of the early history of the condition we are considering, and shall proceed at once to the more distinctive objective symptoms and signs which point to a dilated stomach. The principal symptoms are—(1) a constant bad taste in the mouth, particularly in the morning; (2) restless nights, with sense of oppression in breathing, resembling asthma; and (3) the occasional vomiting of very large quantities of dark-coloured matter, principally fluid. Of these symptoms the first, of course, is not confined to this particular form of indigestion; but I have found it much more constant and objectionable than in other forms. The second has been graphically described by Sir William Broadbent in his recent Harveian lecture, and is a source of great annoyance and even alarm. In cases of slight dilatation it merely causes restless nights, occasionally waking the patient up with a feeling of distension and oppression; but when there is a permanent pool of stagnant and fermenting fluid in the stomach the gradual accumulation of gas, pressing the diaphragm up against the heart and lungs, causes great dyspnœa, and in neurotic subjects often starts a bad attack of asthma. The third symptom, that of vomiting, is very characteristic, the vomited matter varying greatly in appearance and quantity, but being generally dark in colour from the generation of sulphuretted hydrogen, frothy in appearance, and having a very sour odour. If examined microscopically it will often be found to contain the characteristic groups of sarcinæ. The quantity may amount to several pints, and its evacuation gives great but only temporary relief. The most prominent signs are—(1) an increase of the stomach resonance; (2) a slush, which can be best obtained by placing one hand flat on the epigastrium and the other on the left hypochondrium, and pressing alternately with each; (3) loss of flesh; and (4) the presence of permanent residual fluid in the stomach. The resonance, of course, is increased in all directions, but the downward increase is apt to be complicated by the presence of a distended colon, which is difficult to differentiate, although various observers have formulated rules for distinguishing the two. Sometimes, it is true, the outline of the lower edge of the stomach can be plainly seen and even felt by passing the hand gently downwards over the abdomen, but in my experience this is exceptional, and I am accustomed to take the upper limit of resonance as a guide, and find it much more reliable, of course making due allowance for the character of the last meal and the interval which has elapsed. This line of resonance may, and often does, reach as high in the left chest as the fourth rib, and terminates abruptly in the very different note obtained over the lung. (2) The splash which is obtained on succussion is often very characteristic, but in cases of moderate dilatation is not always easily obtained, and its absence does not prove that the stomach is of normal size. (3) Loss of flesh is constant, but varies, of course, with the duration of the case. In long-standing cases it is so marked and alarming as, even in purely functional cases, to suggest the presence of malignant disease. I have seen several such, and the rapidity with which the loss of flesh progressed was sufficient in one case to cause one of the best practitioners in England to give the patient a very few months to live, although with appropriate treatment he soon began to improve, and is now in full and active work. (4.) The last and most certain means of diagnosis consists in the discovery of a permanent collection of fluid in the stomach which is not expelled when that organ has finished its work. This can only be ascertained with certainty by the passage of a tube at an interval of, say, four or six hours after a meal, and the withdrawal of the fluid, after which additional information can be obtained by gently syphoning in plain water until the stomach will hold no more. By this means we are able to find out exactly what the organ is capable of holding. These symptoms and signs are sufficient to establish the diagnosis, and we will now consider the best means of treating the condition.

3. *Treatment.*—When once the fact of dilatation is established, I think it may be laid down as an axiom that the right treatment is to wash the stomach out. It is pure waste of time to pour drugs into the stomach and give peptonised milk, &c., when there is a pool of fermenting fluid there into which everything goes. First wash the cavity out, and then adopt means to gradually restore the power of contractility to its walls. Various appliances have been made to effect this purpose, and of late years they have much improved, but yet this treatment is not nearly so generally adopted as it should be. Anyone who is doing it for the first time will find the following two suggestions useful. Do not use a very large tube, but have one with a large eye, and an opening at the extremity. Have it made entirely of rubber, and no glass tubing or glass funnel, they are always breaking and are no advantage. The large tubes which are usually sold are much thicker and bigger than is necessary, and cause considerable distress on passing through the fauces; the eye which is usually made is a mere slit, and the extremity is solid. This makes it easier to pass, but is really unnecessary, as the tube with an opening at the extremity passes with no difficulty, and is much more effective. The only advantage in having it all in one piece of rubber is that a glass funnel or a piece of glass tubing in the middle is apt to break when carrying it about in a bag, and one is likely to be left in a fix, which never happens with the rubber. Always keep it hanging up to avoid kinks. It is as well to have a mark on the rubber about fifteen inches from the point, in order that you may know when you enter the stomach, although with a large stomach one often has to pass it several inches futher to reach the residual fluid. If on examining the fluid withdrawn sarcinæ are found, it is a good plan to finish the washing out with a pint or two of a solution of hypophosphite of soda, ʒi. to the pint, which retards their further growth. The most agreeable substance with which to grease the tube is a little fresh butter, it leaves no nasty taste behind. The washing out should be continued every day, or every second, third, or fourth, according to the quantity and character of the fluid found at each sitting. I have made no attempt to give directions for making the diagnosis more exact, according to the character of the fluid withdrawn, although it has been stated that the complete absence of free hydrochloric acid points to carcinoma, but this must necessarily be difficult to ascertain in general practice. Of course, the treatment by washing must be supplemented by a carefully selected and, at first, peptonised diet, consisting of thickened or solid food, and at the same time the exhibition of liq. bismuth with nux vomica. These measures will, in a case due to mechanical causes alone, soon bring about improvement, but in the neurotic cases—which comprise by far the larger number—a much longer course of treatment directed to the general health and mode of living will be required.

In conclusion, I will add that, although all cases of dilatation, not due to destruction by a pyloric growth, are greatly benefited by this treatment, yet they are not the only ones so relieved. Cases in which large stringy masses of mucus are removed by the tube generally get much relief from occasional washing, which allays the irritable condition of the gastric walls and the pain which is often experienced.

## Figures and Tables

**Figure f1:**
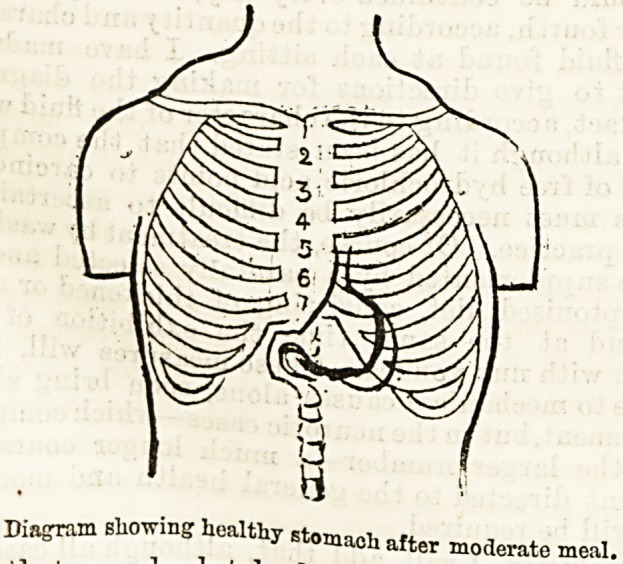


**Figure f2:**